# Clinical Profile of Acute Coronary Syndrome Presentation to the Ladysmith Provincial Hospital: High Prevalence Among the Minority Indian Population

**DOI:** 10.7759/cureus.17670

**Published:** 2021-09-02

**Authors:** Abdullah E Laher, Bonnard E Mumpi, Craig Beringer, Callistus Enyuma, Muhammed Moolla, Feroza Motara

**Affiliations:** 1 Emergency Medicine, University of the Witwatersrand, Johannesburg, ZAF

**Keywords:** acute coronary syndrome, acs, stemi, nstemi, ua, ihd, myocardial infarction, coronary artery disease, migrant indian

## Abstract

Background

Cardiovascular diseases were responsible for 17% of the 460236 natural deaths in South Africa in 2015. Previous studies have reported a disproportionately higher incidence of ischemic heart disease (IHD) and its risk factors among individuals of Indian descent residing in South Africa. The aim of this study was to explore the clinical profile of patients presenting with a diagnosis of acute coronary syndrome (ACS) and to compare the characteristics of patients of Indian descent to those of non-Indian descent.

Methods

Retrospective data were derived from the medical charts of 160 consecutive patients presenting to the Ladysmith Provincial Hospital over a 44-month period with a diagnosis of ACS. Findings were described and compared.

Results

The mean (SD) age of study patients was 55.8 (±12.8) years. The majority of subjects were male (n=90, 56.3%) and unemployed (n=98, 62.3%). The racial distribution of the study sample comprised 103 (64.4%) Indian, 36 (22.5%) Black, and 21 (13.1%) White subjects. Compared to non-Indian subjects, a significantly higher proportion (p<0.05) of Indian subjects were male (64.7% vs 41.4%), cigarette smokers (52.0% vs 32.8%), had a previous history of ACS (37.3% vs 10.3%), were diabetic (33.3% vs 17.2%), and were hypertensive (58.8% vs 29.3%).

Conclusion

The disproportionately high frequency of ACS among the minority Indian population of Ladysmith is concerning. There is a need for rigorous public health interventions to create local awareness, encourage lifestyle modification, and thereby improve control of cardiovascular risk factors, especially among high-risk population groups.

## Introduction

In 2019, ischemic heart disease (IHD) was ranked as the number one cause of global mortality, being responsible for approximately 16% (8.9 million) of total deaths in the world. From the years 2000 to 2019, death from IHD had increased by more than two million cases [1]. Approximately three-quarters of global cardiovascular disease (CVD)-related deaths occur in low- and middle-income countries, with IHD accounting for the majority of these cases [2]. In South Africa, CVD was responsible for 17% (81782) of the 460236 natural deaths in 2015, with IHD accounting for 12224 of these deaths [3]. 

Acute coronary syndrome (ACS) is a constellation of clinical features that results from coronary artery occlusion, which is commonly due to the formation of a thrombus on a ruptured atherosclerotic plaque [4]. The spectrum of ACS includes unstable angina (UA), ST-segment elevation myocardial infarction (STEMI), and non-STEMI (NSTEMI) [5]. Hypertension, diabetes mellitus, dyslipidemia, obesity, smoking, and a sedentary lifestyle are modifiable risk factors that predispose to ACS. Non-modifiable risk factors include genetic factors, age, gender, race, and a lower socioeconomic status [6]. Depending on the profile of ACS presentation, percutaneous coronary intervention (PCI) and thrombolytic therapy (fibrinolysis) are the two currently available therapeutic interventions capable of restoring coronary perfusion [7].

The South African Indian population first arrived in South Africa as migrant laborers around the turn of the twentieth century and settled in the KwaZulu-Natal (KZN) province of the country [8]. Earlier studies conducted in the city of Durban, KZN province reported a disproportionately higher incidence of IHD among individuals of Indian descent compared to those of other racial groups [9-11]. Studies among migrant Indian populations residing in other countries have also reported a disproportionately higher incidence of IHD [12-13]. The city of Ladysmith is situated in the KZN province of South Africa, approximately 250 km inland from the city of Durban. While the above-referenced Durban-based studies were conducted approximately 20-30 years ago, there have been no recent studies pertaining to the presentation of ACS in the Ladysmith region. Hence, the aim of this study was to retrospectively explore the clinical profile of patients that presented to the Ladysmith Provincial Hospital with a diagnosis of ACS and to compare the characteristics of patients of Indian descent to those of non-Indian descent.

## Materials and methods

This study was a cross-sectional retrospective review of the medical records of a convenience sample of 160 consecutive patients that presented to the Ladysmith Provincial Hospital with a diagnosis of ACS between November 1, 2009, and June 31, 2013. Ladysmith Provincial Hospital is a regional and district-level urban public-sector hospital. Of the nine provinces in South Africa, KZN has the second largest population and is ranked third in economic growth [14]. The hospital has 458 beds and serves a population of approximately 26739 people [15]. Ethics clearance for the study was obtained from the Human Research Ethics Committee of the University of the Witwatersrand (clearance certificate M120801) while permission to conduct the study was granted by the hospital management.

For the purpose of this study, the diagnosis of ACS encompassed all patients presenting with a diagnosis of STEMI (including left bundle branch block (LBBB) fulfilling Sgarbossa’s criteria [16]), NSTEMI, and UA as recorded by the attending clinician. Patients with an initial diagnosis of ACS but later reviewed to another diagnosis were excluded from the study. As per internationally accepted practice guidelines, primary PCI, if able to be performed within 120 minutes of STEMI diagnosis, is preferred over thrombolytic therapy [17]. However, since PCI is unavailable at the Ladysmith Provincial Hospital and the duration required to transfer a patient to the closest PCI facility may take longer than 120 minutes, thrombolytic therapy is the first-line treatment of patients with STEMI fulfilling criteria for early reperfusion therapy at the Ladysmith Provincial Hospital. Patients in whom thrombolytic therapy is contraindicated and who may still benefit from emergent PCI are, however, transferred to the appropriate PCI facility.

Comorbidities and risk factors, such as hypercholesterolemia, diabetes mellitus, and renal dysfunction, were captured as present if any of these conditions were recorded as previously diagnosed on patient history, or if the laboratory-measured values for total cholesterol/low-density lipoprotein cholesterol (hypercholesterolemia), glucose (diabetes mellitus), or creatinine/ glomerular filtration rate (renal dysfunction) were elevated above the laboratory defined upper reference limit. Similarly, hypertension was reported as present if either the attending clinician had recorded as previously diagnosed in the patient’s medical records, or if the patient fulfilled the criteria for hypertension as per the latest international hypertension guidelines at the time of data collection [18]. Cardiogenic shock was defined as the presence of a systolic blood pressure <90 mmHg secondary to ACS-related myocardial dysfunction, in the absence of other causes of shock [19].

Prior to the commencement of data collection, the primary investigator underwent informal training pertaining to the methods of data collection from medical records. The primary investigator thereafter reviewed ED registers to identify potential study subjects that had presented with ACS during the data collection period. In addition, intensive care unit, high care unit, and medical ward registers were also reviewed to identify patients that were not listed in the ED register. The medical records of these patients were thereafter obtained from the hospital records department. Patients whose medical records could not be obtained were excluded from the study. Data pertaining to patient demographic details, relevant history and examination findings, presence of ACS risk factors, relevant electrocardiography (ECG) and laboratory findings, treatment instituted, and patient outcomes were collected by the primary investigator. To assess for inter-rater reliability, data from a sample of 25 randomly selected medical records were re-abstracted by an independent individual with previous experience in data abstraction from medical records and who was blinded to the study methodology and the information obtained by the primary investigator. The overall Cohen's kappa coefficient (κ) was 0.86, indicating acceptable inter-rater reliability.

Data were initially entered into a specifically designed data collection sheet and thereafter entered into Microsoft® Excel® (Microsoft 365, Version 16.0.13029.20232, Microsoft Corporation, Redmond, WA) and exported to STATA® version 12 software (StataCorp Limited, Texas) for statistical analyses. The mean and standard deviation were determined for subject age at presentation. All other variables were categorical in nature and described by means of frequency and percentage. Variables were thereafter compared between Indian and non-Indian subjects to determine the presence of statistically significant differences between the two groups. The student t-test was used to compare age at presentation while the remainder of the variables were compared by means of the Pearson’s chi-square test. A two-sided p-value of <0.05 was considered significant for all comparisons. Reporting of findings were in conformance with STROBE (Strengthening the Reporting of Observational Studies in Epidemiology) guidelines [20].

## Results

The names and file numbers of 197 potential study subjects that presented over the 44-month data collection period were retrospectively identified from the respective ED, ICU, HC, and medical ward registers. Of these, the medical records of 29 (14.7%) patients could not be retrieved. A review of the available records identified that the diagnosis was not ACS in eight of the 168 (4.8%) files that were analyzed. Hence, the final study sample comprised 160 subjects with ACS.

Table 1 describes the various characteristics of study subjects and compares the characteristics of Indian subjects with those of non-Indian subjects. The mean (SD) age of study patients was 55.8 (±12.8) years. Majority of subjects were male (n=90, 56.3%) and unemployed (n=98, 62.3%). The racial distribution of the study sample comprised 103 (64.4%) Indian (66 males, 37 females), 36 (22.5%) Black (15 males, 21 females), and 21 (13.1%) White (9 males, 12 females) subjects. Close to half the number of study subjects were cigarette smokers (n=72, 45.0%), hypertensive (n=77, 48.1%), and displayed an elevation in serial troponin I measurements on laboratory analysis (n=72, 45.0%). A quarter (n=40, 25.0%) of study subjects presented with a history of atypical chest pain. Overall, 13 (8.1%) subjects died prior to hospital discharge.

A total of 62 (38.8%) subjects with ST-segment elevation and four (2.5%) subjects with an LBBB pattern fulfilling Sgarbossa’s criteria on ECG were considered for early reperfusion therapy. Of these, thrombolytic therapy was not administered to 23 (34.8%) subjects, as they had presented late and after the recommended cut-off time period. Thrombolytic therapy was administered to 40 (60.6%) of the 66 subjects while three (4.5%) subjects with contraindications to thrombolytic therapy were emergently transferred to the referral PCI facility. Additionally, seven of the 40 (17.5%) subjects who were administered thrombolytic therapy but deemed not to have achieved appropriate STEMI resolution were also emergently transferred for rescue PCI. As per the study facility protocol, besides the subjects that were transferred for emergent PCI, all other study subjects with ACS were also transferred to the referral PCI facility within 24 hours of ED arrival.

Compared to non-Indian subjects, a significantly higher proportion of Indian subjects were male (p =0.007), cigarette smokers (p=0.029), had a previous history of ACS (p=0.005), and were diabetic (p=0.043) and hypertensive (p=0.0006). Compared to non-Indian subjects, Indian subjects had an approximately two-fold higher prevalence of diabetes mellitus (17.2% vs 33.3%) and hypertension (58.8% vs 29.3%) and an approximate three and a half-fold higher prevalence of a previous history of ACS (37.3% vs 10.3%). However, presentation with atypical features of ACS (p=0.006) and mortality (p =0.023) was significantly higher among non-Indian compared to Indian subjects.

**Table 1:** Characteristics of study subjects and comparison between Indian and non-Indian subjects BMI – body mass index; STEMI – ST-segment elevation myocardial infarction; LBBB – left bundle branch block; ECG – electrocardiography

	Entire cohort (n=160)	Indian (n=102)	Non-Indian (n=58)	P-value
Age [mean (SD)] (years)	55.8 (±12.8)	56.1 (±10.7)	55.3 (±15.8)	0.343
Male [n (%)]	90 (56.3)	66 (64.7)	24 (41.4)	0.007
Unemployed [n (%)]	98 (62.3)	64 (62.7)	34 (58.6)	0.729
Alcohol consumption [n (%)]	23 (14.4)	14 (13.7)	9 (15.5)	0.939
Cigarette smoking [n (%)]	72 (45.0)	53 (52.0)	19 (32.8)	0.029
Previous history of ACS [n (%)]	44 (27.5)	38 (37.3)	6 (10.3)	0.0005
Family history of ACS [n (%)]	29 (18.1)	22 (21.6)	7 (12.1)	0.198
Hypertension [n (%)]	77 (48.1)	60 (58.8)	17 (29.3)	0.0006
Hypercholesterolemia [n (%)]	49 (30.6)	36 (35.3)	13 (22.4)	0.128
BMI ≥ 30 [n (%)]	45 (28.1)	23 (22.5)	22 (37.9)	0.058
Diabetes mellitus [n (%)]	44 (27.5)	34 (33.3)	10 (17.2)	0.043
Renal dysfunction [n (%)]	27 (16.9)	22 (21.6)	5 (8.6)	0.060
Atypical chest pain [n (%)]	40 (25.0)	16 (15.7)	24 (41.4)	0.0006
Cardiogenic shock [n (%)]	7 (4.4)	6 (5.9)	1 (1.7)	0.404
STEMI or new LBBB on ECG [n (%)]	66 (41.3)	35 (34.3)	29 (50.0)	0.075
Elevated troponin I at presentation [n (%)]	72 (45.0)	40 (39.2)	32 (55.2)	0.074
Thrombolytic therapy [n (%)]	40 (25.0)	24 (23.5)	16 (27.5)	0.704
Mortality [n (%)]	13 (8.1)	4 (3.9)	9 (15.5)	0.023

## Discussion

The prevalence of ACS in this study was 51.5 patients per annum, which is more than double the mean number of average cases reported (19.8 per annum per facility) in a Canadian study in 2006 across 53 large-volume hospitals [21]. This is concerning and is an area that requires further research to investigate factors contributing to the overall relatively high prevalence of ACS at the study site.

With regards to risk factors for ACS, 45% of study subjects were smokers. Comparatively, other studies reported rates of between 41% and 63% [22-24]. Hypertension and hypercholesterolemia were present in 48.1% and 30.6% of study subjects, respectively. Comparatively, these comorbidities were respectively present in 34%-75% and 6%-59% of patients in other similar studies [6,23,25]. Obesity (BMI ≥ 30) was present in 28.1% of study subjects, which is similar to 25% reported by El-Menyar et al. in the Gulf RACE study [25]. Similar to the ACCESS (23.9%) [22] and GRACE (24.3%) [26] studies, diabetes mellitus was present in 27.5% of study subjects, while renal dysfunction was present in 16.9% of subjects, which was similar to findings of two other studies [25,27].

In this study, 75.0% of patients presented with typical chest pain, which is similar to the figures reported by Cakir and Blue (70.8%) [28] and El-Menyar et al. (83%) [25]. However, Brieger et al. reported a much lower incidence of 33.3% [26]. Similar to findings of other studies [25-26], 4.4% of subjects in this study presented with cardiogenic shock. Less than half (41.3%) the number of study subjects presented with STEMI or a presumably new LBBB on ECG, which is similar to the 41% reported in the ACCESS study [22] and 40% reported in a study by de Lemos et al. that included data from 41 countries [23]. However, other studies reported much lower rates of 12.7% [29] and 15% [24]. Compared to other studies that reported an in-hospital mortality rate of between 3.27% and 6.9% [25-27,30], the overall in-hospital mortality rate was slightly higher at 8.1% in this study.

As per the 2011 national census, the racial distribution of the Ladysmith region comprises 91.8% Black, 4.4% Indian, 2.7% White, and 1.0% Colored (mixed race) citizens [31]. Although individuals of Indian descent comprise only 4.4% of the Ladysmith population [32], approximately two-thirds of study subjects were of Indian descent (64.4%) with approximately two-thirds of them being male. Cigarette smoking and hypertension were significantly more common among Indian subjects (p<0.05). A cross-sectional study comprising 1428 adults from the suburb of Phoenix in Durban documented an extremely high prevalence of cardiovascular risk factors that included hypertension (47.5%), hypercholesterolemia (47.5%), diabetes mellitus (20.1%), and smoking (>50% of males) [33]. A study by Seedat et al. that was conducted in the metropolitan area of Durban and enrolled 778 subjects of Indian descent reported that the prevalence of smoking was 60.2%, hypertension 46.1%, hypercholesterolemia 56.1%, and diabetes mellitus 15.8% [9]. Another Durban-based study by Ranjith et al. that recruited 245 young South African Indians subjects (≤ 45 years of age) presenting with a diagnosis of acute myocardial infarction reported that the prevalence of smoking was 74%, hypertension 22%, hypertriglyceridemia 19%, and diabetes mellitus 54% while only 14% of patients were female [11]. Comparatively, in the current study, the prevalence of smoking was 45.0%, hypertension 48.1%, and diabetes mellitus 27.5% while only 56.3% of patients were male. A study conducted in the United States concluded that first-generation immigrant Indian men had a higher prevalence of coronary heart disease, non-insulin-dependent diabetes mellitus, low HDL levels, and hypertriglyceridemia [34]. Another study conducted in London, UK, also reported that diabetes mellitus, hypertension, high serum insulin levels, low HDL cholesterol levels, and high plasma triglyceride levels were more prevalent in South Asian Indian immigrants than those of European descent [12]. Yet another study conducted in Riyadh, Saudi Arabia, reported that the incidence of ACS was two-fold higher among immigrant South Asians, who were also younger in age, had a lower socioeconomic status, and had higher rates of cigarette smoking [35].

Despite the disproportionately higher prevalence of ACS among Indian subjects in this study, mortality was significantly higher among non-Indian subjects (p=0.023). The higher prevalence of atypical chest pain presentation among non-Indian subjects (p=0.0006), is a likely reason for the higher mortality in this group. Other studies have also reported that an atypical presentation of ACS was associated with higher mortality [36-37].

From the above, it is evident that the findings of our study are of grave concern. There is a need to conduct similar studies among Indian population groups in other regions of the country as well as in other countries. There is also a need to initiate awareness, develop health screening initiatives, and organize educational programs with a view to encouraging lifestyle modification and improving cardiovascular risk factor control, especially among high-risk population groups. 

A retrospective analysis holds less weight than a prospective study. As such, this study is subject to known limitations pertaining to spurious findings, missing data, and conflicting data. Moreover, this was a single-center study conducted in a public hospital, hence our findings may not be applicable to the private setting or to other geographic parts of South Africa. Also, because of the nature of the presentation of ACS, especially in its atypical form, clinicians may have inadvertently missed the diagnosis of ACS, resulting in underdiagnosis and under-reporting of the actual prevalence of ACS.

The prevalence of ACS among the Indian population of Ladysmith is disproportionately high. There is a need for further public health research among migrant Indian population groups in other regions. There is also a need to initiate awareness, develop health screening initiatives, and organize educational programs with a view to encouraging lifestyle modification and improving cardiovascular risk factor control, especially among high-risk population groups.

## Conclusions

The prevalence of ACS among the Indian population of Ladysmith is disproportionately high. There is a need for further public health research among migrant Indian population groups in other regions. There is also a need to initiate awareness, develop health screening initiatives, and organize educational programs with a view to encouraging lifestyle modification and improving cardiovascular risk factor control, especially among high-risk population groups.
